# The role of repeat cerclage in managing subsequent pregnancies for patients with a prior history of cervical cerclage: a retrospective self-control study

**DOI:** 10.3389/fmed.2025.1544075

**Published:** 2025-04-07

**Authors:** Lidan Zhang, Mengyang Hu, Xiaofu Yang, Peng Zhao

**Affiliations:** Department of Obstetrics, Women’s Hospital, Zhejiang University School of Medicine, Hangzhou, China

**Keywords:** cervical cerclage, cervical insufficiency, repeat cerclage, initial cerclage, preterm birth

## Abstract

In a woman who received cerclage in the previous pregnancy, obstetricians were highly likely to perform this surgery in her subsequent pregnancy. However, many researchers have advocated against the use of repeat cerclage. The objective of this study was to evaluate the effectiveness of repeat cerclage in managing the subsequent pregnancy for participants with a history of cervical cerclage. We retrospectively collected data from patients who had a history of cervical cerclage and received repeat cerclage in the subsequent pregnancy. A self-controlled comparative analysis was undertaken to evaluate the differences in baseline characteristics and pregnancy outcomes between the initial cerclage and the repeat cerclage. A total of 173 patients were included in the study. These patients were divided into two groups, the initial cerclage group and the repeat cerclage group. Consequently, the gestational age at delivery, birth weight, live birth outcome, and neonatal morbidity in the repeat cerclage group were significantly improved compared to the initial cerclage group (*p* < 0.001 for all aforementioned indicators). All patients were further divided into four subgroups based on their indications for initial cerclage. Specifically, 54 patients received an initial cerclage due to prior history (group A), 45 patients based on ultrasound findings (group B), 63 patients due to physical examination (group C), and 11 participants for inappropriate indications (group D). As a result, repeat cerclage significantly increased both gestational age at delivery and birth weight in group A, group B, and group C, with statistical significance noted as follows: group A (*p* = 0.007 for gestational age, *p* = 0.044 for birth weight), group B (*p* = 0.002 for gestational age, *p* = 0.011 for birth weight), and group C (*p* < 0.001 for both) No significant differences were noted in group D. In conclusion, the clinical outcome of repeat cerclage in patients with a prior history of cervical cerclage, regardless of whether it was indicated by history, ultrasound, or physical examination, was found to be significantly beneficial for the patients. For patients who have undergone a prior cerclage based on evidence-supported indications of CI, repeat cerclage may be a prudent consideration.

## Introduction

Preterm birth (PTB) and its associated complications constitute the primary cause of mortality and disability among perinatal infants and children under 5 years of age, resulting in a total of 1 million deaths annually (1). Cervical insufficiency (CI) is one of the significant risk factors leading to PTB (2). It is documented that CI complicates approximately 1% of all pregnancies, accounting for 8% of participants who experience recurrent mid-trimester pregnancy loss (3). Cervical cerclage has been established as an effective measure for preventing PTB in pregnancies with CI. Cerclage can be warranted by three key indicators: history-indicated CI, ultrasound-indicated CI, and physical-examination indicated CI (4). Once a woman has undergone a cervical cerclage in her previous pregnancy, she is highly likely to require a repeat cerclage in her subsequent pregnancy. In such cases, obstetricians typically make the decision to perform this surgery based on the patients’ prior surgical history and her individual preference. However, the aforementioned strategy remains controversial. Many researchers have advocated against the use of repeat cerclage for patients without a clinical history of painless cervical dilation in the second trimester of pregnancy (5, 6), especially for those whose diagnosis of CI was solely based on ultrasound findings (7). Pelham et al. (5) reported that for patients with uncertain indications for a previous cerclage, repeat cerclage may not lead to improved clinical outcomes. On the other hand, Vousden et al. (7) advocated that for patients with a history of ultrasound-indicated cerclage, ultrasound monitoring could be a preferred approach for those who do not require abdominal cerclage during their subsequent pregnancy. However, these perspectives have not yet gained widespread acceptance. We assumed that the controversy arises primarily from interindividual differences inherent in the design of the above-mentioned studies. Specifically, one study (6), though self-controlled, suffered from a small number of participants, whereas the remaining two were retrospective cohort studies (5, 7) that lacked self-control. When we study a repeat procedure, such as repeat cerclage, the most important factor influencing the clinical outcome is inter-individual variability. This is because each individual patient possesses unique physiological, anatomical, and medical characteristics that can significantly impact how they respond to a repeated intervention (8–12). Factors such as pregnancy history, previous surgical outcomes, surgical skills, postoperative management, and any underlying health conditions can all contribute to this variability. Given the complexity of inter-individual variabilities, we are firmly convinced that a rigorously designed self-controlled study, coupled with a substantial number of participants, is the most appropriate approach to effectively manage and address these variations. We believe that repeat cerclage in the subsequent pregnancy could improve gestational age at delivery and neonatal outcomes during clinical practice. Therefore, we conducted the current self-control study to evaluate the effectiveness of repeat cerclage in managing the subsequent pregnancy for patients with a history of cervical cerclage.

## Methods

All participants diagnosed with CI were delivered at Women’s Hospital, Zhejiang University School of Medicine, from July 1991 to December 2023. The medical records were obtained and reviewed thoroughly and finally confirmed by two obstetricians to ensure accuracy. CI was diagnosed as an inability of the uterine cervix to retain a pregnancy in the second trimester in the absence of labor signs (13). Cervical cerclage was performed in cases where patients had a history of three or more spontaneous second-trimester losses and/or PTBs (history indicated) or exhibited advanced painless cervical dilation without labor and abruptio placentae in the second trimester (physical examination indicated), or had a short cervical length in the second trimester coupled with a history of one or more spontaneous second-trimester losses or PTBs (ultrasound-indicated) (2, 14, 15). Participants were enrolled if they met the following criteria: (1) they were diagnosed with CI; (2) it was retrospectively confirmed that they had undergone a repeat cerclage procedure during their subsequent pregnancies, following a prior history of cervical cerclage; and (3) they were singleton pregnancies. Participants were excluded if they had multiple pregnancies, were diagnosed with preterm rupture of the fetal membranes prior to the cerclage procedure (due to its status as a surgical contraindication for cerclage) or chorioamnionitis prior to the cerclage procedure, or had missing data. The current study was approved by the ethics committee of the authors’ institution.

The cervical cerclage was exclusively completed using a transvaginal approach. The type of cerclage, either Shirodkar or McDonald, was determined and conducted by experienced senior obstetricians with either Mersilene tape or conventional non-absorbable surgical sutures.

Data pertaining to baseline characteristics (including maternal age, body mass index (BMI), gravidity, parity, and mode of conception), indications for cervical cerclage and surgery details (such as the technique employed, gestational age at placement), and pregnancy outcomes (encompassing gestational age at delivery, birth outcomes, birth weight, Apgar score, NICU admission, duration of NICU stay, neonatal morbidity, maternal morbidity, and postpartum hemorrhage) were collected. A self-controlled comparative analysis was undertaken to evaluate the differences between the initial cerclage and the repeat cerclage, enabling a thorough assessment of their respective effects and outcomes.

To reduce selection bias, we identified eligible patients based on predefined criteria and systematically reviewed their records to avoid subjective selection. Additionally, we implemented abstraction protocols to ensure consistency and objectivity in case identification and inclusion. Regarding recall bias, we took several precautions. First, we relied on documented medical records, rather than solely on patient recall, as these records provide a more objective and consistent source of information. Moreover, where patient recall was necessary, we employed standardized protocols to collect data, which helped minimize variations in responses.

Statistical analysis was performed with the SPSS package (version 20) for Microsoft Windows (IBM Corp., Armonk, NY, United States). This self-controlled study encompassed both continuous and categorical variables. Differences between groups were compared using a paired Student’s *t*-test for continuous variables (such as gestational age at delivery and birth weight) and a paired chi-square test for categorical variables. Subgroup analysis was performed according to the indications of the initial cerclage. A *p*-value <0.05 was considered statistically significant.

## Results

### Participants

**Figure 1:**
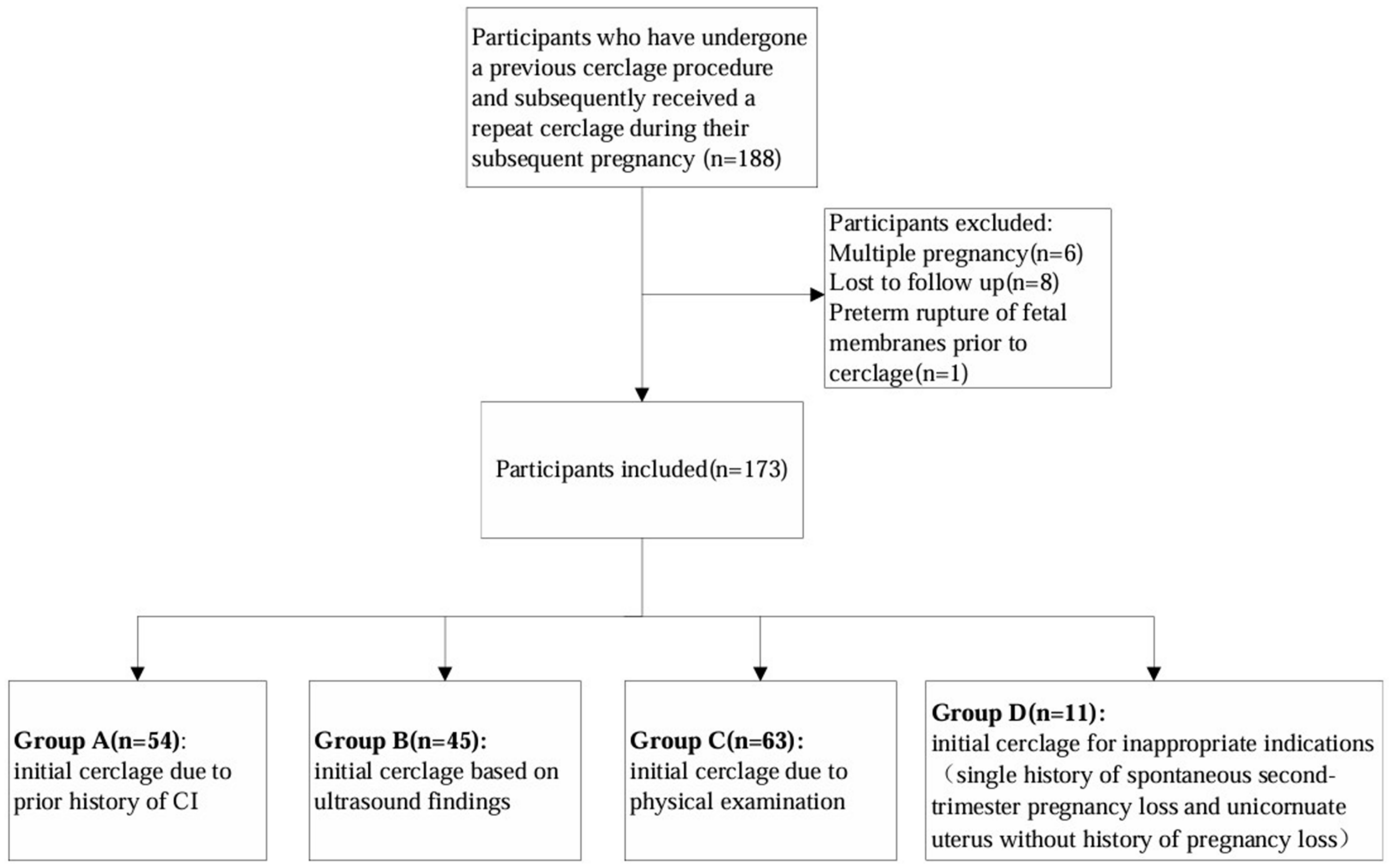
Flow diagram.

A total of 188 patients who had a documented history of cervical cerclage and underwent the same procedure during their subsequent pregnancy were enrolled in this study. Fifteen patients were excluded due to multiple pregnancies, loss to follow-up, and preterm rupture of the fetal membranes prior to cerclage, and a total of 173 patients were finally included in the study. The results are presented as follows (Figure 1).

### Clinical characteristics

All of the included patients were divided into two groups: the initial cerclage group, serving as the control group, and the repeat cerclage group, designated as the study group. The clinical characteristics of the patients in both groups are presented in Table 1. Consequently, the patients in the repeat cerclage group demonstrated a significantly higher maternal age, gravidity, and parity, along with a notably lower gestational age at placement when compared with the initial cerclage group (initial versus repeat: mean maternal age: 29.10 versus 32.22, *p* < 0.001; mean gravidity: 2.66 versus 3.82, *p* < 0.001; mean parity: 0.15 versus 0.81, *p* < 0.001, mean gestational age at placement: 18.50 versus 14.87, *p* < 0.001, respectively). In terms of mode of conception, the patients in the repeat cerclage group showed a significantly lower prevalence of assisted reproductive technology utilization (initial versus repeat: 21.97% versus 14.45%, *p* = 0.029). No significant differences were noted pertaining to BMI and the cerclage technique.

**Table 1:** The clinical characteristics of the participants.

Characteristics	Initial cerclage	Repeat cerclage	*p*-value
Maternal age (years)	29.10 ± 3.98	32.22 ± 4.17	<0.001
BMI	23.93 ± 4.02	24.03 ± 3.71	0.629
Gravidity	2.66 ± 1.46	3.82 ± 1.58	<0.001
Parity	0.15 ± 0.37	0.81 ± 0.60	<0.001
Gestational age at placement (weeks)	18.50 ± 4.47	14.87 ± 2.97	<0.001
Mode of conception			
Unassisted	135 (78.03%)	148 (85.55%)	
Ovulation induction	10 (5.78%)	1 (0.58%)	
Artificial insemination	0	3 (1.73%)	
*In vitro* fertilization	28 (16.18%)	21 (12.14%)	0.029
Cerclage technique			
McDonald	152 (87.86%)	144 (83.24%)	
Shirodkar	10 (5.78%)	25 (14.45%)	
Details unattainable*	11 (6.36%)	4 (2.31%)	0.981

BMI, body mass index; SD, standard deviation. Data were presented as mean ± SD/n (%). *, The details of the cervical cerclage procedure were unattainable due to the fact that the surgery was not performed at our hospital.

### Primary pregnancy outcomes

The comparison of clinical outcomes is presented in Table 2. As a result, the gestational age at delivery in the repeat cerclage group was significantly elevated than that in the initial cerclage group (initial versus repeat: 31.53 ± 7.37 weeks versus 35.84 ± 4.55 weeks, *p* < 0.001). Similarly, the birth weight and live birth outcome in the repeat cerclage group were notably increased (initial versus repeat: 2308.68 ± 1248.11 versus 2928.71 ± 881.65, *p* < 0.001; 65.90% versus 94.22%, *p* < 0.001, respectively).

**Table 2:** Pregnancy outcomes.

Outcomes	Initial cerclage	Repeat cerclage	*p*-value
Gestational age at delivery (weeks)	31.53 ± 7.37	35.84 ± 4.55	<0.001
Mode of delivery			
Caesarean section	44 (25.43%)	89 (51.45%)	
Vaginal delivery	129 (74.57%)	84 (48.55%)	<0.001
Birth outcomes			
Live birth	114 (65.90%)	163 (94.22%)	
Stillbirth	59 (34.10%)	10 (5.78%)	<0.001
Birth weight (g)	2308.68 ± 1248.11	2928.71 ± 881.65	<0.001
Apgar score			
1st minute	9.55 ± 1.40	9.72 ± 0.93	0.307
5th minute	9.79 ± 1.53	9.92 ± 0.34	0.442
Postpartum hemorrhage			
<500 mL	151 (87.28%)	159 (91.91%)	
≥500 mL	22 (12.72%)	14 (8.09%)	0.131
NICU admission*			
Yes	41 (35.96%)	49 (30.06%)	
No	73 (64.04%)	114 (69.94%)	0.046
Time (days) spent in NICU	5.99 ± 11.78	5.22 ± 14.28	0.660
Neonatal morbidity			
Yes	92 (53.18%)	49 (28.32%)	
No	81 (46.82%)	124 (71.68%)	<0.001
Maternal morbidity			
Yes	64 (36.99%)	91 (52.60%)	
No	109 (63.01%)	82 (47.40%)	<0.001

Abbreviations: NICU, neonatal intensive care unit; SD, standard deviation. Data are presented as mean ± SD/n (%). *, In the initial cerclage group, 59 patients had either a miscarriage or stillbirth, subsequently leading to the absence of data on NICU admission. Similarly, in the repeat cerclage group, 10 patients experienced a miscarriage or stillbirth, resulting in no information being recorded on NICU admission.

### Secondary pregnancy outcomes

The incidence of NICU admission and neonatal morbidity in the repeat cerclage group was significantly lower (initial versus repeat: 35.96% versus 30.06%, *p* = 0.046; 53.18% versus 28.32%, *p* < 0.001). However, a significantly increased incidence of Caesarean section and maternal morbidity was noted in the repeat cerclage group (initial versus repeat: 25.43% versus 51.45%, *p* < 0.001; 36.99% versus 52.60%, *p* < 0.001, respectively). No significant differences were noted in the incidence of postpartum hemorrhage, Apgar score, and time spent in the NICU. Specifically, regarding maternal morbidity, our study revealed significantly higher rates of gestational diabetes (29.48% vs. 11.56%, *p* < 0.001), thyroid disorders (9.24% vs. 3.47%, *p* = 0.018), and autoimmune diseases (6.94% vs. 3.47%, *p* = 0.014) in the repeat cerclage group than the initial cerclage group. No statistically significant differences (*p* > 0.05) were noted in cervical laceration, premature rupture of membranes, gestational hypertension, chorioamnionitis, placental abruption, intrahepatic cholestasis of pregnancy, or cardiac arrhythmia, with *p*-values ranging from 0.071 to 0.852. Details are presented in Table 3.

**Table 3:** Comparison of maternal morbidity between two groups.

Maternal complications	Initial cerclage	Repeat cerclage	*p*-value
Gestational diabetes			
Yes	20 (11.56%)	51 (29.48%)	
No	153 (88.44%)	122 (70.52%)	<0.001
Thyroid disorders			
Yes	6 (3.47%)	16 (9.24%)	
No	167 (96.53%)	157 (90.75%)	0.018
Autoimmune disease			
Yes	6 (3.47%)	12 (6.94%)	
No	167 (96.53%)	161 (93.06%)	0.014
Cervical laceration			
Yes	0	1 (0.58%)	
No	173 (100.00%)	172 (99.42%)	0.317
Premature rupture of membrane			
Yes	24 (13.87%)	25 (14.45%)	
No	149 (86.13%)	148 (85.55%)	0.852
Gestational hypertension			
Yes	10 (5.78%)	15 (8.67%)	
No	163 (94.22%)	158 (91.33%)	0.275
Chorioamnionitis			
Yes	12 (6.94%)	5 (2.89%)	
No	161 (93.06%)	168 (97.11%)	0.071
Placental abruption			
Yes	4 (2.31%)	6 (3.47%)	
No	169 (97.69%)	167 (96.53%)	0.480
Intrahepatic cholestasis of pregnancy			
Yes	7 (4.05%)	9 (5.20%)	
No	166 (95.95%)	164 (94.80%)	0.480
Cardiac arrhythmia			
Yes	0	1 (0.58%)	
No	173 (100.00%)	172 (99.42%)	0.317

Data are presented as *n* (%).

### Subgroup analysis of pregnancy outcomes

Based on the clinical practice guidelines of cervical cerclage (2, 14, 15), all participants were divided into four subgroups based on their indications for initial cerclage. Group A included participants who received initial cerclage due to history-indicated CI, group B comprised those who were based on ultrasound findings, group C consisted of those who were due to physical examination, and group D encompassed those who underwent initial cerclage for inappropriate indications. Consequently, there were 54 participants recruited for group A, 45 participants for group B, and 63 participants for group C. A total of 11 participants were assigned to Group D, with 10 of them having a single medical history of painless cervical dilation leading to second-trimester pregnancy loss, and the remaining one being diagnosed with a unicornuate uterus without a preceding history of pregnancy loss. Subgroup analysis was conducted to compare gestational age at delivery and birth weight between initial cerclage and repeat cerclage. The results showed that, when compared to initial cerclage, repeat cerclage significantly increased both gestational age at delivery and birth weight in group A (initial versus repeat: gestational age at delivery: 32.26 ± 6.94 versus 34.89 ± 4.86, *p* = 0.007; birth weight: 2394.59 ± 1179.21 versus 2750.00 ± 892.60, *p* = 0.044), group B (initial versus repeat: gestational age at delivery: 32.73 ± 8.14 versus 36.38 ± 4.84, *p* = 0.002; birth weight: 2531.88 ± 1275.25 versus 3115.13 ± 942.41, *p* = 0.011), and group C (initial versus repeat: gestational age at delivery: 28.98 ± 6.82 versus 35.90 ± 4.22, *p* < 0.001; birth weight: 1858.63 ± 1233.63 versus 2870.20 ± 812.60, *p* < 0.001). However, no significant differences were noted in group D (initial versus repeat: gestational age at delivery: 37.55 ± 2.95 versus 38.00 ± 2.57, *p* = 0.320; birth weight: 3200.91 ± 772.33 versus 3318.18 ± 768.14, *p* = 0.379). Data are presented in Table 4.

**Table 4:** Subgroup analysis of pregnancy outcomes based on indications for initial cerclage.

Outcomes (*n* = 173)	Initial cerclage	Repeat cerclage	*p*-value	95% CI for pairwise comparison
Gestational age at delivery (weeks)				
Group A (*n* = 54)	32.26 ± 6.94	34.89 ± 4.86	0.007	−4.51, −0.74
Group B (*n* = 45)	32.73 ± 8.14	36.38 ± 4.84	0.002	−5.82, −1.47
Group C (*n* = 63)	28.98 ± 6.82	35.90 ± 4.22	<0.001	−8.73, −5.11
Group D (*n* = 11)	37.55 ± 2.95	38.00 ± 2.57	0.320	−1.42, 0.51
Birth weight (g)				
Group A (*n* = 54)	2394.59 ± 1179.21	2750.00 ± 892.60	0.044	−701.14, −9.67
Group B (*n* = 45)	2531.88 ± 1275.25	3115.13 ± 942.41	0.011	−1022.83, −143.67
Group C (*n* = 63)	1858.63 ± 1233.63	2870.20 ± 812.60	<0.001	−1396.00, −627.13
Group D (*n* = 11)	3200.91 ± 772.33	3318.18 ± 768.14	0.379	−401.43, 166.88

1. Data are presented as mean ± SD. 2. Group A: initial cerclage due to a prior history of cervical incompetence; group B: initial cerclage based on ultrasound findings; group C: initial cerclage due to physical examination; group D: initial cerclage for inappropriate indications (single history of spontaneous second-trimester pregnancy loss and unicornuate uterus without history of pregnancy loss).

## Discussion

The major findings of our study revealed that for patients with a prior history of cervical cerclage, regardless of whether it was indicated by history, ultrasound, or physical examination, repeat cerclage in their subsequent pregnancies was associated with significantly improved primary pregnancy outcomes, including a notably increased gestational age at delivery and birth weight and a decreased probability of secondary outcomes such as neonatal morbidity and NICU admission.

Two methods of transvaginal cerclage placement were described by Shirodkar and McDonald in the 1950s (16). Since then, many researchers have demonstrated that cervical cerclage was effective in promoting pregnancy outcomes for patients with CI (4, 16, 17). However, the decision to repeat or not to repeat cervical cerclage has been a major source of confusion for clinicians when managing patients with a previous history of cerclage (5). Some researchers (6) advocated repeat cerclage exclusively for patients who had a prior cerclage based on the classic history of pregnancy loss due to painless mid-term miscarriage. While other researchers believed that repeat cerclage in the subsequent pregnancy did not improve pregnancy outcomes in patients with a prior history-indicated cerclage or those with ultrasound-indicated cerclage (18). For instance, Vousden et al. (7) investigated a total of 54 patients who had undergone ultrasound-indicated cerclage in their previous pregnancies. Of these patients, 23 received repeat cerclage, 23 were monitored using transvaginal ultrasound, and 8 patients underwent transabdominal cerclage. The study revealed that, for patients who had previously undergone ultrasound-indicated cerclage, ultrasound monitoring, rather than repeat cerclage, during the subsequent pregnancy, emerged as a viable and suitable option. Moreover, another study, reported by Suhag et al. (19) in 2015, involved a larger number of patients with a history of ultrasound-indicated cerclage. This research included 102 singleton pregnancies, of which 38 patients (37.3%) underwent ultrasound surveillance, whereas 64 patients (62.7%) received prophylactic repeat cerclage in their subsequent pregnancies. Consequently, primary pregnancy outcomes (gestational age at delivery and/or birth weight) were found to be comparable between the two groups and the authors concluded that both transvaginal ultrasound cervical length screening and repeat cerclage were acceptable options. The findings of these two studies suggested that for patients who had previously undergone ultrasound-indicated cerclage, repeating the procedure in a subsequent pregnancy did not lead to improved clinical outcomes. Consequently, they proposed that ultrasound monitoring might serve as a more suitable alternative in such cases. Nevertheless, our results contradict this conclusion. The disparity of available evidence posed a dilemma for clinicians in dealing with a pregnant woman who had a cerclage. We attributed the inconsistency of the above-mentioned studies to the limited number of participants and the confounding effects of differences between individuals. To address this issue, we deemed a self-control study with a larger sample size as an effective approach and, accordingly, embarked on this research endeavor as our current focus. With this approach, we are confident that our study offered a more robust and reliable solution.

The decision-making process for clinicians regarding whether to proceed with a repeat cerclage was exceedingly complex in patients who receive an initial cerclage for indications not adhering to evidence-based data, particularly if their prior obstetric outcomes were deemed favorable, because one might be inclined to opt for a repeat surgery due to the patient’s insistence or pressure. To address this perplexing issue, we conducted an in-depth subgroup investigation focusing on this specific cohort of patients, summarizing the pertinent data in Table 4, designated as group D. Our findings revealed that, in group D, the gestational weeks at delivery of this cohort were 37.55 weeks, a duration that was considered satisfactory. However, when these patients underwent a subsequent cerclage procedure, their average gestational weeks at delivery increased slightly to 38.00 weeks, and statistical analysis failed to show significant differences (*p* = 0.320). Our results demonstrated that in patients who have undergone prior cerclage for indications other than a history-indicated, ultrasound-indicated, or physical examination-indicated CI, repeat cerclage did not yield a notable improvement. Our results were consistent with a previous study by Pelham et al. (5). In their study, a total of 56 patients with a history of cerclage for non-traditional indications were included. One group of 28 patients received repeat cerclage, and the parallel group of 28 patients was monitored by transvaginal ultrasound. As a result, there were no differences in the primary pregnancy outcomes (gestational age at delivery, PTB < 35 weeks, and spontaneous PTB < 35 weeks) between groups. They concluded that, in such cases, repeat cerclage may not be an effective intervention.

It is noteworthy that maternal morbidity, particularly gestational diabetes, thyroid disorders, and autoimmune diseases, was significantly higher in the repeat cerclage group than in the initial cerclage group. This disparity may be attributed to the advanced maternal age in the repeat cerclage group (32.22 ± 4.17 years) versus the initial cerclage group (29.10 ± 3.98 years, *p* < 0.001). Previous studies have established that advanced maternal age is associated with increased risks of gestational diabetes (20), thyroid disorders (21), and autoimmune diseases (22).

Based on the findings of our study, the clinical outcome of repeat cerclage in patients with a prior history of cervical cerclage, when performed based on evidence-supported indications, was found to be significantly beneficial for the patients. Consequently, repeat cerclage appears to be a promising option for these patients, as it demonstrates a favorable outcome in terms of clinical benefit.

Our study has several strengths. First, as a rigorous self-controlled study, comparisons are conducted within individuals, effectively eliminating all time-invariant confounding factors, ensuring the precision and validity of the findings. Second, the current study stands out with an unprecedentedly larger sample size, empowering a thorough and robust comparison of pregnancy outcomes. Third, by conducting tailored subgroup analyses specific to the indications for initial cerclage, the study achieves a heightened level of detail and grants a profound understanding of the intricate factors that influence pregnancy outcomes. However, it is essential to acknowledge that the retrospective nature of our study posed challenges in terms of data completeness and the potential for bias (such as lack of long-term neonatal outcomes and potential unmeasured confounders).

## Conclusion

In conclusion, repeat cerclage may be a prudent consideration for patients who received prior cerclage based on evidence-supported indications (classic history of CI, ultrasound findings, or physical examination). Drawing upon our findings, physicians can make well-informed decisions pertaining to the appropriateness of repeat cerclage for individual patients. However, because of the respective nature of our study, further multicentered randomized controlled trials need to be conducted to confirm our results.

## Data Availability

The original contributions presented in the study are included in the article/supplementary material, further inquiries can be directed to the corresponding author.
